# The Ship Movement Trajectory Prediction Algorithm Using Navigational Data Fusion

**DOI:** 10.3390/s17061432

**Published:** 2017-06-20

**Authors:** Piotr Borkowski

**Affiliations:** Maritime University of Szczecin, Wały Chrobrego 1, Szczecin 70500, Poland; p.borkowski@am.szczecin.pl

**Keywords:** prediction of ship movement trajectory, navigational data fusion, navigational decision support system

## Abstract

It is essential for the marine navigator conducting maneuvers of his ship at sea to know future positions of himself and target ships in a specific time span to effectively solve collision situations. This article presents an algorithm of ship movement trajectory prediction, which, through data fusion, takes into account measurements of the ship’s current position from a number of doubled autonomous devices. This increases the reliability and accuracy of prediction. The algorithm has been implemented in NAVDEC, a navigation decision support system and practically used on board ships.

## 1. Introduction

It has been assumed herein that the term “navigational data fusion” (unlike the broader concept of navigational data integration [1,2,3,4,5,6,7]) is a fusion of data from different, duplicated sources, (navigational measuring instruments) describing the same physical quantity. The data obtained through fusion are based on information from all of the available sensors. Consequently, such data are characterized by greater precision than those from each separate sensor. In such cases the system safety level is enhanced, because in the absence of data from a specific source (device failure) the other still guarantees effective operation. This is particularly important in real-time systems. Data fusion, therefore, provides more reliable and more accurate information.

Practice shows that most sea-going vessels have autonomous position determining devices doubled. The navigator dealing with data on their own ship position from various sources may face difficulties in predicting the trajectory of their own ship and making the right decisions. On the other hand, relying on measurements from a single device only is an unreliable solution with a risk of vital information loss. It, therefore, seems justified to make use of the data fusion mechanism for navigational data prediction and synthesis.

The proposed navigational data fusion process is based on a multi-sensor Kalman filter. The filtered signals are weighted by a filtration error cross-covariance matrix, then the averaged output signal is calculated. This signal may be used in the further process of predicting a ship movement trajectory over a specified time.

The developed computing algorithm, in order to minimize prediction errors, makes use of artificial neural networks (like in [8,9,10,11]), trained by the adaptive method. This approach consists of using a large number of neural networks taught from data strings of different lengths. The network that learn from short data strings permitting better approximate movement parameters of a maneuvering ship. The function of the networks learning from longer data strings is the approximation of a steady movement (non-maneuvering vessels). Values of predicted movement parameters are determined based on the weighted responses of each network. It has been adopted that the values of weights for each network response are calculated on the basis of prediction errors and current values of the ship movement obtained through the navigational data fusion. This approach is effective for predicting trajectories of other ships [12,13]. For one’s own ship we can assume that its future course alterations and the actual parameters of the ship dynamics model are known, which significantly improves the reliability of prediction [14]. It is proposed that the prediction of one’s own ship trajectory will be obtained as a result of simulated autopilot operation, involving an LQR regulator and a linear ship movement dynamics model [15,16].

The methods of ship movement trajectory prediction presented in the literature are often difficult to implement in practice. The reasons for this are limitations of these methods [17,18,19,20,21] or the complexity of the adopted hydrodynamic model, on which the prediction is based [22,23,24]. Such a model must be created individually for each ship and, additionally, there is need to identify model parameters for different navigational conditions, which is an extremely difficult task. That is why the navigational systems installed on the ship’s bridge mostly use the prediction based on the extrapolation of current traffic parameters (speed, acceleration), assuming that these parameters will not change significantly in the future [25]. These methods are burdened with a large error and in most cases serve for rough estimation of the ship’s future position. The proposed algorithm of ship trajectory prediction using the navigational data fusion process is a solution with high accuracy, offering a possibility of its effective implementation in marine navigational systems.

## 2. The Navigational Data Fusion

The fusion of navigation data obtained from measurements carried out by *l* sensors is expressed by the weighted average [26,27]:

(1) $$\left[\begin{array}{c} \tilde{x}\left(t\right)\\ \tilde{y}\left(t\right)\\ {\tilde{v}}_{x}\left(t\right)\\ {\tilde{v}}_{y}\left(t\right) \end{array}\right]=A_{1}\left(t\right)\cdot \left[\begin{array}{c} {\hat{x}}_{1}\left(t\right)\\ {\hat{y}}_{1}\left(t\right)\\ {\hat{v}}_{x1}\left(t\right)\\ {\hat{v}}_{y1}\left(t\right) \end{array}\right]+A_{2}\left(t\right)\cdot \left[\begin{array}{c} {\hat{x}}_{2}\left(t\right)\\ {\hat{y}}_{2}\left(t\right)\\ {\hat{v}}_{x2}\left(t\right)\\ {\hat{v}}_{y2}\left(t\right) \end{array}\right]+\ldots +A_{l}\left(t\right)\cdot \left[\begin{array}{c} {\hat{x}}_{l}\left(t\right)\\ {\hat{y}}_{l}\left(t\right)\\ {\hat{v}}_{xl}\left(t\right)\\ {\hat{v}}_{yl}\left(t\right) \end{array}\right]$$

where:

$\left(\tilde{x}\left(t\right),\tilde{y}\left(t\right)\right)$ fusion of Cartesian coordinates estimates of vessel position at an instant *t*;
$\left({\tilde{v}}_{x}\left(t\right),{\tilde{v}}_{y}\left(t\right)\right)$ fusion of vessel speed estimates in the reference system at an instant *t*;
$A_{i}\left(t\right)$ fusion weights’ matrices;
$\left({\hat{x}}_{i}\left(t\right),{\hat{y}}_{i}\left(t\right)\right)$ estimates of Cartesian coordinates of vessel position at an instant *t* determined using a Kalman filter for a measurement by the *i*-th sensor (1≤i≤l); and
$\left({\hat{v}}_{xi}\left(t\right),{\hat{v}}_{yi}\left(t\right)\right)$ estimates of vessel speed in the reference system at an instant *t* determined using a Kalman filter for a measurement by the *i*-th sensor (1≤i≤l).

Navigational data fusion weights matrices are determined from the formula [26,27]:

(2) $$A_{i}\left(t\right)={\left[\sum_{j=1}^{l}{P_{jj}}^{-1}\left(t\right)\right]}^{-1}\cdot {P_{ii}}^{-1}\left(t\right)$$

where:

$P_{ij}\left(t\right)$ matrix of cross-covariance of filtration errors between *i*-th and *j*-th sensor-specific subsystems.

Matrices of filtration error cross covariance are determined from this formula [26,27]:

(3) $$P_{ij}\left(t\right)=\left[I_{4}-K_{i}\left(t\right)\cdot H\right]\cdot \left[\Phi \cdot P_{ij}\left(t-1\right)\cdot \Phi^{T}\right]\cdot {\left[I_{4}-K_{j}\left(t\right)\cdot H\right]}^{T}$$

where:

$I_{4}$ 4 × 4 unit matrix;
$K_{i}\left(t\right)\;\left(K_{j}\left(t\right)\right)$ is the matrix of the Kalman filter gain at an instant *t* for *i*-th (*j*-th) sensor-specific subsystem (1≤i≤l,1≤j≤l) [26,27]; and
H,Φ constant matrices of the system: $H=\left[\begin{array}{cccc} 1 & 0 & 0 & 0\\ 0 & 1 & 0 & 0 \end{array}\right]$, $\Phi =\left[\begin{array}{cccc} 1 & 0 & 1 & 0\\ 0 & 1 & 0 & 1\\ 0 & 0 & 1 & 0\\ 0 & 0 & 0 & 1 \end{array}\right]$.

Defined by Equations (1)–(3), the navigational data fusion process is optimal, because it minimizes the trace of the fusion estimator error matrix [28].

The vector of navigational data fusion may be used in the further process of predicting the ship trajectory for a specified time span. This procedure increases the reliability and accuracy of prediction.

## 3. Prediction of other Ship Trajectories

One way of predicting the movement trajectory of a ship, i.e., regarded as a complex, dynamic, technical system, is the application of artificial neural networks. They make up a universal approximating system, mapping multidimensional datasets. They have an ability to learn and adapt to changing environmental conditions. They are also capable of generalizing in various areas, including the prediction of time series and the identification of control objects. They are an interesting alternative to traditional methods in the absence of a precise hydrodynamic model of the ship and in the case of difficulties and time constraints relating to the identification of analytical model parameters. Their speed of operation makes them applicable in real-time systems.

Typical applications of artificial neural networks for identification, prediction, and control require that a proper training set should be gathered. The training process is a one-time procedure based on the collected data that should include all situations likely to occur.

An alternative method would be the construction of adaptive systems, in which training processes take place repeatedly along with changing movement conditions. The adaptive approach is used in the proposed method of prediction of other ship movement parameters. This approach employs a large number of neural networks trained by data strings of different lengths. Networks that learn from short data strings permit better approximate movement parameters of manoeuvring ships. The function of networks trained from longer data strings is the approximation in the case of steady movement (ships that are not maneuvering). Values of predicted movement parameters are determined based on weighted responses of individual networks.

This approach eliminates the need to identify a manoeuvre (as is the case in solutions using two networks: for a manoeuvring ship and a ship not performing any rudder and/or engine manoeuvers). By changing the weights the system smoothly changes the predictions: for steady movement (no manoeuvring), or for a manoeuvring ship. In this connection it is important to properly assign weights to the responses of the particular networks. It was assumed that the values of weights for each network (prediction of movement parameters) are calculated based on prediction errors, which, in turn, are based on the current values of ship movements obtained via the navigational data fusion of Equations (1)–(3):

(4) $$w_{i}=\frac{\varepsilon_{\min}+\varepsilon_{\max}-\varepsilon_{i}}{m\varepsilon_{\min}+m\varepsilon_{\max}-\sum_{i=1}^{n}\varepsilon_{i}}$$

where:

*m* number of neural networks;
*i* numeral of a network (1≤i≤m);
$w_{i}$ weight for the *i*-th network response;
$\varepsilon_{i}$ *i*-th network prediction error;
$\varepsilon_{\min}$ minimum value of the prediction error made by a network; and
$\varepsilon_{\max}$ maximum value of the prediction error made by a network.

The algorithm of other ship movement parameter prediction is as follows:

1. Recording of training data strings using the process of navigation data fusion (Equations (1)–(3)).
2. Training of GRNN networks.
3. Determination of the prediction errors of the networks on the basis of the current values of ship movement parameters using the navigational data fusion process (Equations (1)–(3)).
4. Determination of the weights for each network response.
5. Determination of the network response for the current values of the movement parameters using the navigational data fusion process (Equations (1)–(3)).
6. Determination of the values of predicted movement parameters based on weighted responses of each network.

The algorithm makes use of the generalized regression neural network (GRNN) with a structure containing four functionally-diversified layers: input, radial, regression, and output. The number of radial neurons is equal to the number of training data. The regression layer consists of two neurons, the output layer has one. Responses of the radial neuron layer are multiplied by values of desired responses, and the result is divided by the sum of the radial neuron responses. Radial neurons in regression networks may be trained using any self-learning technique, while the regression and output layers are trained by means of a specialized algorithm [29].

The proposed algorithm can be used for predicting the trajectory of both one’s own and another ship. However, if we have a hydrodynamic model of one’s own ship, its use in the prediction process is highly justified.

## 4. Prediction of One’s Own Ship Trajectory

For the purposes of predicting the trajectory of one’s own ship, an LQR regulator was used, similarly to the problem of course stabilization. This is possible if the ship model parameters, as well as planned course alterations, are known. This assumption is true for one’s own ship.

The usual practice in designing algorithms for a complex dynamic object is an application of a simplified, mostly linear model, which makes up a basis for further synthesis of the system. Let the dynamics of a continuous stationary object be described by a linear equation of state:

(5) $$\dot{x}=Ax+Bu$$

where:

A a matrix with the size n×n and fixed elements independent of time;
B a matrix with the size n×p and fixed elements independent of time;
X *n-dimensional* vector of state;
$\dot{x}$ *n-dimensional* vector of derivatives versus time (*t*); and
u *p-dimensional* vector of control signals.

with the initial condition:

(6) $$x\left(0\right)=x_{0}$$

while the control quality indicator has this form:

(7) $$J\left(u\right)=\int_{0}^{\infty }\left(x^{T}R_{x}x+u^{T}R_{u}u\right)dt$$

where:

$R_{x}$ symmetric, positive semidefinite matrix, with dimensions n×n and fixed elements being independent of time; and
$R_{u}$ symmetric, positive definite matrix, with dimensions p×p and fixed elements being independent of time.

The problem to be considered is the determination of such a control vector $u^{*}$ that will meet Equation (5) and minimize the quality criterion defined by Equation (7). Optimally, the control takes this form:

(8) $$u^{*}={R_{u}}^{-1}B^{T}Px$$

where matrix **P** is determined from the algebraic Riccati’s equation:

(9) $$0=-PA-A^{T}P-PB{R_{u}}^{-1}B^{T}P+R_{x}$$

where:

0 zero matrix with dimensions n×n.

Let the model describing the dynamics of ship movement will be a Nomoto model [16] including a course deviation as a state component. Then, to be consistent with the form of Equation (5), the following denotations are made:

(10) $$\begin{array}{cccc} A=\left[\begin{array}{cc} 0 & 1\\ 0 & -a \end{array}\right];\; & B=\left[\begin{array}{c} 0\\ c \end{array}\right];\; & x=\left[\begin{array}{c} \psi \\ r \end{array}\right];\; & u=\left[\delta \right] \end{array}$$

where:

ψ deviation from the vessel’s course;
$r=\dot{\psi }$ rate of turn;
δ rudder angle; and
a,c parameters determined from ship model tests, always assumed to be known for one’s own ship.

The control quality in the problem of ship course stabilization is mostly defined by this indicator [16]:

(11) $$\int_{0}^{\infty }\left(\psi^{2}+\lambda \delta \right)dt$$

where:

λ a coefficient greater than zero, which is interpreted as a compromise between course deviation (yaw angle) and rudder angle (steering gear load);

that, after introducing the matrices:

(12) $$\begin{array}{cc} R_{x}=\left[\begin{array}{cc} 1 & 0\\ 0 & 0 \end{array}\right];\; & R_{u}=\left[\lambda \right] \end{array}$$

will take the form of Equation (7).

In the case of data: Equations (10) and (12) and the algebraic Riccati Equation (9) will lead to the following equations (taking into account the symmetry of matrix **P**):

(13) $$\begin{array}{ccc} -\frac{1}{\lambda }c^{2}{p_{12}}^{2}+1=0;\; & -\frac{1}{\lambda }c^{2}p_{12}p_{22}-p_{11}+ap_{12}=0;\; & -\frac{1}{\lambda }c^{2}{p_{22}}^{2}-2p_{12}+2ap_{22}=0 \end{array}$$

where:

$p_{ij}$ element of matrix **P** in the *i*-th row and *j*-th column;

After its solution, we obtain:

(14) $$\begin{array}{l} p_{11}=\pm \sqrt{a^{2}+\frac{2c}{\sqrt{\lambda }}}\\ p_{12}=-\frac{\sqrt{\lambda }}{c}\\ p_{22}=\frac{\sqrt{\lambda }}{c^{2}}\left(a\pm \sqrt{a^{2}+\frac{2c}{\sqrt{\lambda }}}\right) \end{array}\;\mathrm{lub}\;\begin{array}{l} p_{11}=\pm \sqrt{a^{2}-\frac{2c}{\sqrt{\lambda }}}\\ p_{12}=\frac{\sqrt{\lambda }}{c}\\ p_{22}=\frac{\sqrt{\lambda }}{c^{2}}\left(a\pm \sqrt{a^{2}-\frac{2c}{\sqrt{\lambda }}}\right) \end{array}$$

Given that the parameter *c* may exceed the value of the expression $\frac{a^{2}\sqrt{\lambda }}{2}$ and bearing in mind the negative determination of the matrix **P**, out of the four solutions of Equation (14) we obtain:

(15) $$P=\left[\begin{array}{cc} -\sqrt{a^{2}+\frac{2c}{\sqrt{\lambda }}} & -\frac{\sqrt{\lambda }}{c}\\ -\frac{\sqrt{\lambda }}{c} & \frac{\sqrt{\lambda }}{c^{2}}\left(a-\sqrt{a^{2}+\frac{2c}{\sqrt{\lambda }}}\right) \end{array}\right]$$

Therefore, for the data: Equations (10), (12), and (15) and the control law (Equation (8)) will take the form:

(16) $$\delta =k_{\psi }\psi +k_{r}r$$

where:

$$\begin{array}{l} k_{\psi }=-\frac{1}{\sqrt{\lambda }}\\ k_{r}=\frac{1}{c}\left(a-\sqrt{a^{2}+\frac{2c}{\sqrt{\lambda }}}\right) \end{array}$$

Thanks to the assumption that parameters *a*, *c* are known, and so are future course alterations (course deviations) ψ, thereby knowing the rate of turn *r*, the control law of Equation (16) may be adopted as the main module of the algorithm for own ship trajectory prediction. The coefficient is λ, determined arbitrarily.

The algorithm of one’s own ship trajectory prediction, apart from the module of control decision-making and the model of dynamics described by Equations (5) and (10), must also take into account the ship’s movement kinematics model. To this end the following is assumed:

(17) $$\begin{array}{c} \dot{x}=u\sin\psi +v\cos\psi \\ \dot{y}=u\cos\psi -v\sin\psi \end{array}$$

where:

(x,y) Cartesian coordinates (ship’s position);
u ship’s longitudinal speed; and
v transverse speed of the ship.

The model described by Equations (5) and (10) should also take into account the dynamics of the steering gear, which is an executive element with constraints on the rudder angle and rate of rudder deflection changes. We can do it by adding Equation (16) to the model dynamics:

(18) $$\dot{\delta }=\delta_{z}-\delta$$

where $\delta_{z}$ is the rudder angle setting.

The relationships between Equations (5), (10), and (16)–(18) constitute one’s own ship trajectory prediction algorithm (for which model parameters and future course alterations are known). The prediction is obtained as a result of the simulation of the course autopilot that operates based on the LQR regulator and linear model of ship movement dynamics. The algorithm takes into account measurement data obtained through the process of the fusion of Equations (1)–(3).

## 5. Implementation of the Proposed Algorithm in the Navigational Decision Support System NAVDEC

The proposed ship trajectory prediction algorithm (for one’s own and other ships) using the navigational data fusion process has been implemented in the navigational decision support system NAVDEC [30,31].

**Figure 1:**
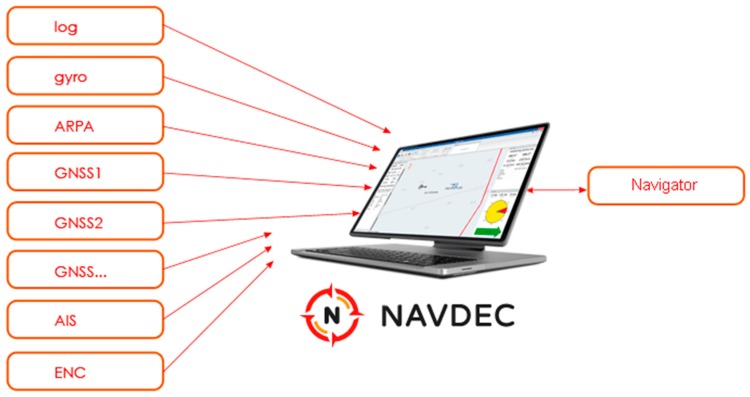
A diagram of the NAVDEC environment [32].

The NAVDEC system is an addition and extension of the navigational bridge equipment onboard a ship. It is a real-time system operated by the navigator. To function properly, NAVDEC has to interact with standard shipboard equipment and systems: log, gyrocompass, ARPA (automatic radar plotting aid), GNSS (global navigational satellite system), AIS (automatic identification system), and ENC (electronic navigational chart), which deliver current navigational data (Figure 1).

The NAVDEC system, like other navigation systems, has information and presentation functions. However, NAVDEC’s innovative functionality is an analysis of a navigational situation taking into account the Collision Regulations, principles of good sea practice, and the criteria used by expert navigators. Where the situation is classified as a collision situation, the system generates suggested anti-collision manoeuvres. The collision situation is presented to the navigator in the form of a rosette, depicting the ranges of allowable courses, i.e., courses that enable one’s own ship to pass all observed targets at a preset safe distance. The rosette also shows a recommended course. The solutions proposed by the system, along with their justification, do not relieve the navigator from responsibility, but facilitate them to take the right decision.

The NAVDEC system has been certified by the Polish Register of Shipping (certification no. TC/2026/901301/15), a globally-recognized classification society, and implemented on ships of different operators (e.g., Polferries).

**Figure 2:**
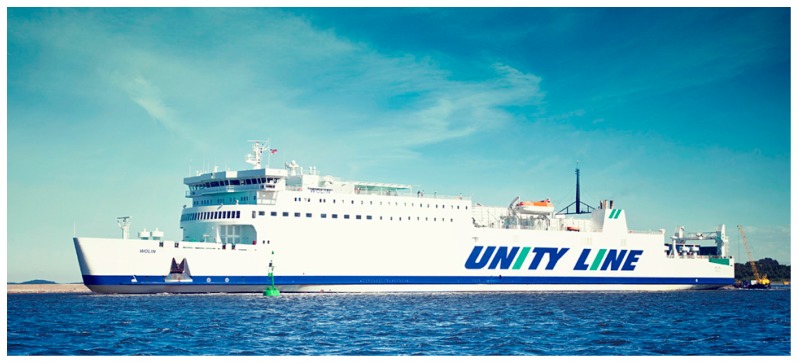
The ferry Wolin—the site of the computing experiments in real conditions [33].

Computing experiments were carried out in the real environment on board M/F Wolin (Figure 2), the largest car/passenger ferry trading in the Baltic. These parameters were adopted as model coefficients: a=0.023[1/s], $c=0.003\left[1/s^{2}\right]$. The selected ship has the following characteristics: maximum rudder angle $\delta_{\max}=35\;\left[{}^{\circ}\right]$, maximum rudder rate of turn ${\dot{\delta }}_{\max}=3.5\;\left[{}^{\circ}/s\right]$. It was established arbitrarily that λ=1.

The prediction of other ships’ trajectories was based on three neural networks trained by properly prepared data strings (data obtained from fusion) with lengths, respectively, of 5, 10, and 15 elements.

**Figure 3:**
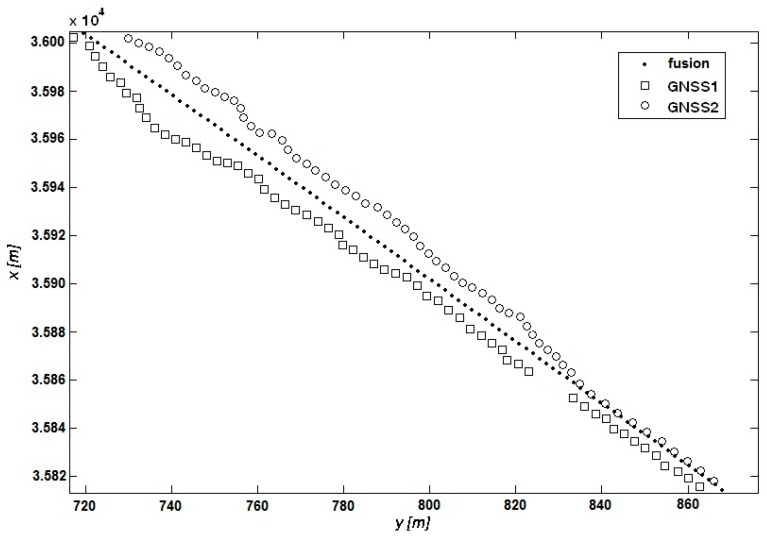
Measurements of ship’s position by two GNSS receivers and the data resulting from fusion.

Figure 3 illustrates examples of ship position data recorded by the GNSS receivers, where measurements were made on a straight section of the track. In order to use the navigational data fusion process Equations (1)–(3) geographic coordinates were transformed into Cartesian coordinates (*x*, *y*) using Gauss-Krüger projection [34]. Notably, the measurements from the two GNSS receivers are not on a straight line, while the data resulting from fusion lie approximately on a straight line. This proves that the navigational data fusion works correctly.

In Figure 3 a situation may also be noted where at some point there is a loss of signal from the GNSS1 receiver. The fusion signal is maintained thanks to the measurements from the GNSS2 receiver. It is essential from the viewpoint of the navigational decision support system, as signal loss can lead to system destabilization and invalid prediction of the ship’s trajectory.

The presented situations are typical of the research done. Not a single case was observed where the measurement data fusion would produce ‘worse’ results than the autonomoous signals from the GNSS receivers.

**Figure 4:**
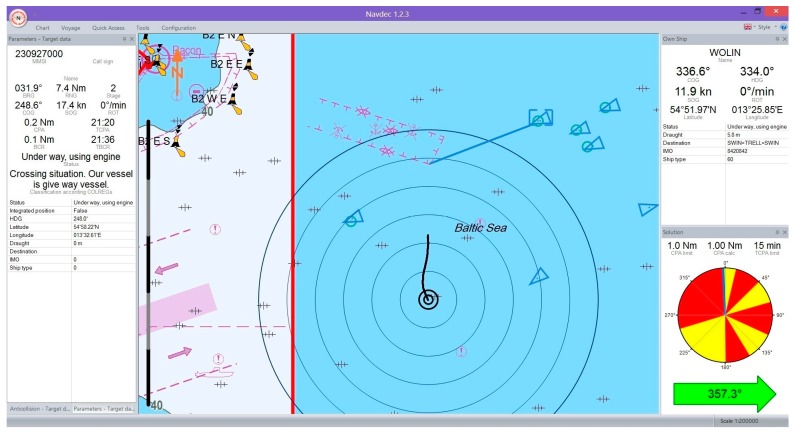
The ship trajectory prediction in the NAVDEC—example one.

**Figure 5:**
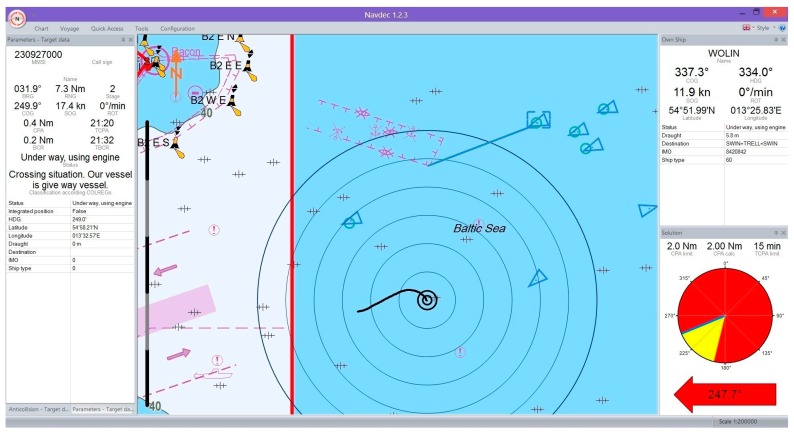
The ship trajectory prediction in the NAVDEC system—example two.

Figure 4 and Figure 5 illustrate example visualizations of own ship trajectory prediction (black line) and a selected other ship (blue line) in the NAVDEC interface. The time span of prediction was set to 15 min. A step alteration of own ship course from the present course to that recommended by the NAVDEC 357.3° 247.7° system was always executed the moment the prediction was displayed. The NAVDEC recommendation depended on the Closest Point of Approach (CPA) setting. In the former case the CPA was 1 Nm; in the latter, 2 Nm.

Table 1 lists the root mean square errors (RMSE) of prediction implemented through the proposed algorithm (without and with the use of the navigational data fusion) and by the Transas ECDIS [35]. The time span of prediction was set to 6 min. The table content confirms greater accuracy of prediction made on the basis of the proposed algorithm than the prediction obtained by the Transas ECDIS (the error is about 80% smaller). The study also confirms that:

- the use of the navigational data fusion process increases the accuracy of prediction,
- prediction for one’s own ship is more accurate than the prediction for a target.

The presented situations are typical of the research done. In all examined cases the prediction of ship movement trajectory was performed correctly. The accuracy of the proposed prediction is significantly higher than the commonly used prediction based on the extrapolation of current ship movement parameters that assumes that the ship movement conditions will not significantly change (unchanged settings of engines, thrusters, rudders, and unchanged external conditions—the parameters of the water area, wind, current, etc.).

## 6. Conclusions

Analysis of the marine accident investigating bodies’ decisions indicates that human error is one of the main causes of accidents at sea. Elimination or reduction of human error, thereby assuring the highest possible level of navigational safety, may be achieved by introducing navigational decision support systems onboard ships. NAVDEC is one of the innovative systems of that type. An important function of the NAVDEC of vital importance to proper assessment and decision-making by the navigator is the reliable and accurate prediction of one’s own ship and target ships’ trajectories within a specified time.

This article presents an algorithm of ship movement trajectory prediction, which, through navigational data fusion, makes use of measurements of the ship’s current position from a number of doubled autonomous devices. In addition, the algorithm takes into account the assumption of knowledge of both future course alterations and the parameters of the ship dynamics model, which takes place in the case of one’s own ship. This increases the reliability and accuracy of prediction, which is desirable in the navigational decision support system. The research carried out under real conditions verifies the proper operation the proposed algorithm and confirms the implementation of the results presented herein.

## Figures and Tables

**Table 1:** The table of root mean square errors of prediction based on the methods compared.

The Method of Prediction	Own/Other Ship	RMSE [m]
Transas ECDIS	not applicable	173
proposed algorithm	other	85
	own	21
proposed algorithm with the data fusion process	other	78
	own	19
